# Finite Element Analysis of Fatigue in Silicon Nitride Ball Bearings Under Hertzian Contact and Lubrication Effects

**DOI:** 10.3390/ma19132856

**Published:** 2026-07-03

**Authors:** Thomas Singleton, Zulfiqar Ahmad Khan, Adil Saeed, Yonggang Meng

**Affiliations:** 1 NanoCorr Energy and Modelling Research Group, Bournemouth University, Poole BH12 5BB, UKzkhan@bournemouth.ac.uk (Z.A.K.); 2 State Key Laboratory of Tribology in Advanced Equipment, Tsinghua University, Beijing 100084, China; mengyg@tsinghua.edu.cn; 3 Department of Mechanical Engineering, New Uzbekistan University, Tashkent 100007, Uzbekistan

**Keywords:** bearings, silicon nitride, hertzian contact, finite element analysis, friction

## Abstract

**Highlights:**

- A finite element framework was developed for Si_3_N_4_ bearing fatigue prediction.
- Lubrication and friction effects were investigated under Hertzian contact.
- Fatigue failure was predicted only under high-friction conditions.
- Low-friction lubrication can maintain stress below the fatigue limit.
- The method can reduce reliance on time-consuming fatigue testing.
- Higher Hertzian pressures and lubricant-specific friction data are recommended.

**Abstract:**

Bearings are essential components in mechanical systems, with ceramic ball bearings increasingly adopted in turbine, automotive, and aerospace applications due to their superior strength and durability. Despite these advantages, bearings are subjected to significant cyclic loading, which can accelerate plastic deformation and lead to sudden catastrophic failure. Current approaches for predicting bearing lifespan rely on time-consuming theoretical and experimental methods. This study proposes a more efficient finite element (FE) approach to predict fatigue behaviour in silicon nitride ball bearings operating under lubricated conditions. In this research, a 12.7 mm diameter silicon nitride ball bearing was analysed under a Hertzian contact pressure of 3 GPa using SolidWorks Simulation 2023 (SWS). Friction coefficients ranging from 0.00 to 1.00 were investigated to represent different lubrication conditions. The results indicate that the stress amplitude remained below the fatigue limit of 1.02 GPa for friction coefficients up to 0.80, while fatigue failure was predicted at a coefficient of 1.00, corresponding to 1.086 × 10^4^ cycles.

## 1. Introduction

The interaction between contact mechanics, lubrication, surface topography, temperature, and material behaviour governs the tribological response of ceramic bearings. Bearing contacts may operate within hydrodynamic, mixed, or boundary lubrication regimes, each characterised by distinct levels of surface separation and friction [1,2,3]. Under hydrodynamic lubrication, a continuous lubricant film completely separates the contacting surfaces, minimising friction and wear. Mixed lubrication occurs when the lubricant film thickness becomes comparable to the composite surface roughness, resulting in intermittent asperity interactions. In contrast, boundary lubrication is characterised by significant asperity contact, elevated friction coefficients, and increased local stress concentrations that accelerate damage accumulation [1,2,3,4,5]. Recent studies have highlighted the growing importance of thin-film and boundary lubrication modelling in rolling-contact fatigue prediction, particularly as bearing systems operate at increasingly higher power densities and contact pressures [6]. Consequently, lubrication regime transitions play a fundamental role in determining bearing reliability and fatigue performance.

Surface topography further influences lubricant film formation and rolling-contact fatigue behaviour. The ratio between lubricant film thickness and composite surface roughness governs the transition between lubrication regimes and directly affects contact stress distributions. Increasing arithmetic surface roughness (Ra) reduces the effectiveness of the lubricant film, promotes asperity interactions, and increases the likelihood of localised crack initiation [1,7]. Previous investigations have demonstrated that wear and fatigue behaviour in silicon nitride (Si_3_N_4_) rolling contacts are strongly dependent on surface condition, with local geometric changes influencing contact pressure distributions, tensile stresses, and crack propagation trajectories [8]. Consequently, surface finish remains a critical design parameter for extending bearing life.

In practical bearing systems, pure rolling conditions rarely exist. Instead, rolling–sliding interactions arise due to creep, differential surface velocities, and kinematic mismatches within the contact zone. These effects modify lubricant entrainment, traction behaviour, frictional energy dissipation, and subsurface stress distributions [2,7]. Experimental studies conducted under rolling–sliding conditions have shown that increasing sliding severity promotes adhesive wear, abrasive wear, delamination, and rolling-contact fatigue damage, underscoring the importance of accounting for rolling–sliding kinematics in bearing-life prediction models [7,9]. Such findings indicate that sliding velocity within Hertzian contacts can significantly influence both wear mechanisms and fatigue crack initiation.

Temperature is another critical parameter influencing tribological performance. Frictional energy generated within concentrated Hertzian contacts produces localised heating that can alter lubricant viscosity, modify elasto-hydrodynamic film formation, accelerate tribochemical reactions, and influence surface oxide layer development [1,3]. Recent thermal analyses of full-ceramic bearings have shown that temperature distributions can significantly affect bearing performance, particularly under starved lubrication or high-speed operating conditions [10]. Furthermore, elevated temperatures may alter local stress distributions and accelerate degradation mechanisms, thereby contributing to premature fatigue failure. Despite these recognised effects, thermo-mechanical coupling remains insufficiently represented in many existing ceramic bearing fatigue models.

In addition to fatigue crack propagation, surface degradation mechanisms contribute significantly to bearing failure. Under inadequate lubrication conditions, direct asperity interactions may result in adhesive wear, material transfer, and localised surface damage [4]. Experimental investigations have shown that adhesive wear frequently precedes contact fatigue and pitting damage under rolling–sliding conditions, particularly at elevated loads [4,7]. These wear mechanisms generate stress concentrations that subsequently act as crack initiation sites, establishing a direct relationship between lubrication quality, frictional behaviour, wear evolution, and rolling-contact fatigue life [11].

## 2. Background Research

### 2.1. Composition of Modern Bearings

The necessity for high-quality bearings has significantly increased over the past decade due to growing demand for turbine, machining, automotive and aerospace applications [12]. These mechanisms subject bearings to high-speed, high-temperature, and heavily lubricated environments, which subsequently inflict higher Hertzian contact stresses [13,14]. Thus, recent technological developments have increased the necessity for high-quality bearings. To manufacture these advanced systems, engineers have developed a series of hybrid ceramics that can resist complex loading.

A common material choice, and the one studied by this report, is silicon nitride (Si_3_N_4_). Silicon nitride is further classified according to the processing techniques: sintered, hot-pressed, reaction-bonded, and hot-isostatically pressed (HIP). HIP Si_3_N_4_ offers a zero-porosity fine-grain microstructure held together by strong covalent bonds, optimising the strength, hardness, toughness and fatigue properties, making it ideal for ball bearing applications [13]. Khan even suggests HIP materials show a greater magnitude of improvement in calculated life.

Most bearing assemblies are also submerged in a lubricated environment, which coats the components with an oil film, separating the solid bodies and therefore preventing asperity contacts between the race and roller [13]. Since the film thickness is dependent on fluid properties, it is safe to assume that the type of lubricant strongly influences the coefficient of friction within the system. Recent scholars have even suggested that complementary lubricants react with the outer surface of the bearing, generating a unique film that further inhibits friction [1,3].

### 2.2. Fatigue Failure of Ball Bearings

Unfortunately, even with advanced mitigations, consecutively applied amplitude stresses cause cumulative damage to the material. In particular, the dynamic loading causes dislocation motions that induce irreversible plastic deformation, which then accumulates over corresponding stress cycles until failure occurs [5,15,16,17]. In the case of Si_3_N_4_, failure commonly occurs due to crack propagation, with microscopic material imperfections expanding over consecutive stress cycles when the stress intensity factor exceeds the fracture toughness of the material [5,7,12,16], eventually leading to fatigue failure and the ultimate breakdown of machines.

### 2.3. Related Experiments

Currently, ball bearing design relies heavily on the theoretical knowledge of fatigue life and subsequent analytical and experimental techniques [5,16,18], which include the Weibull distribution method, fracture mechanics, and rotary tribometers. In general, fracture mechanics provide conservative results but are limited by varying parameters which change per application, whilst experimental procedures offer good estimates but are costly and time-consuming, as large sample sizes must be tested until fatigue spalling occurs [13,19]. It is therefore a common consensus that an efficient life prediction methodology for ceramic ball bearings needs to be developed [12,13,19]. Some advancements towards a computational methodology have been made in the form of finite element analysis (FEA) [15,16,19]. This research provides an effective methodology for predicting fatigue in Si_3_N_4_ bearings, whilst detailing comprehensive instructions to serve as a foundation for industry.

## 3. Materials and Methods

### Experimental Technique

**Figure 1:**
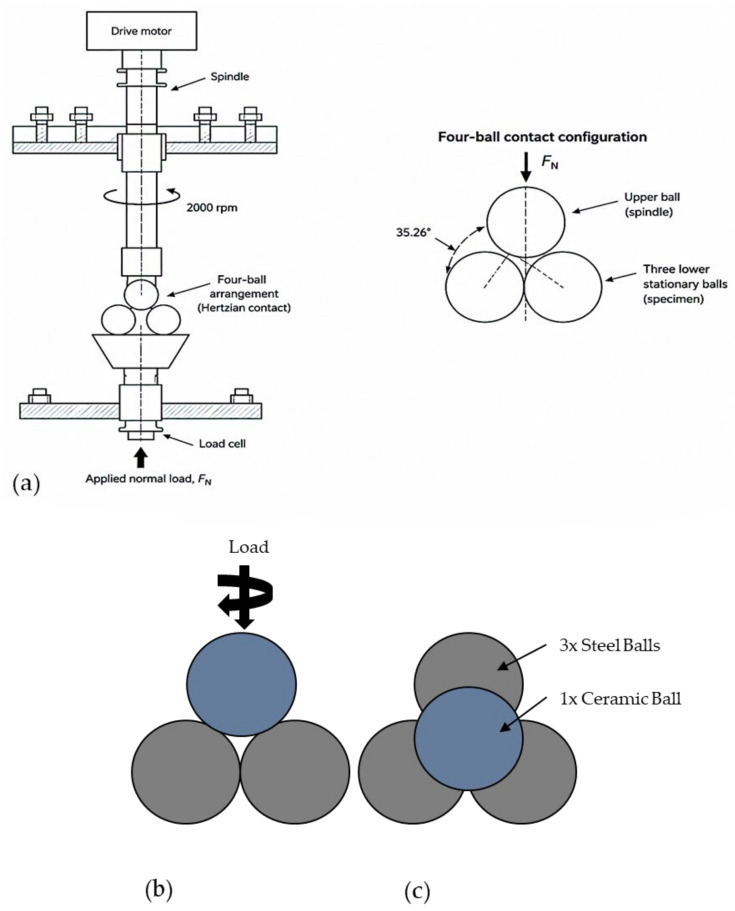
(**a**) Four-ball tribometer schematics, (**b**) four-ball arrangement front view and (**c**) four-ball Hertzian contact configuration.

Four-ball rotary tribometers, shown in Figure 1, reproduce rolling-contact fatigue conditions by generating concentrated Hertzian contacts between rolling elements under controlled loads, rotational speeds, lubrication conditions, and contact stresses. The arrangement closely replicates the contact mechanics, lubrication behaviour, and cyclic loading experienced by rolling-element bearings in service [18].

Furthermore, the four-ball tribometer configuration enables accelerated fatigue testing while maintaining representative Hertzian contact pressures and rolling-contact conditions.

This study utilises experimental results previously reported by Khan [13], in which testing was conducted within a sealed, pressurised chamber containing R600a (isobutane) refrigerant lubricant to replicate refrigeration-system operating conditions. The tribological response of the contact is influenced by the thermophysical properties of the lubricant, particularly its temperature-dependent viscosity, which governs lubricant film formation and frictional behaviour within the contact region [6,13].

The spindle rotational speed was fixed at 2000 rpm (Figure 1) and was converted to a corresponding approximate rolling surface velocity of 1.33 M s^−1^ as below for the 12.7 mm diameter Si_3_N_4_ ball [13].

v = ωr(1)

ω = 2πN/60(2)

Therefore, ω = (2π × 2000)/60 = 209.44 rad/s and v = 209.44 × 0.00635 = 1.33 [m/s].

Thus, the contact operated at an approximate rolling velocity of 1.33 m/s.

The principal experimental conditions and corresponding outcomes adopted in the present finite element investigation are summarised in Table 1.

## 4. Results

### 4.1. Theoretical Calculations

**Figure 2:**
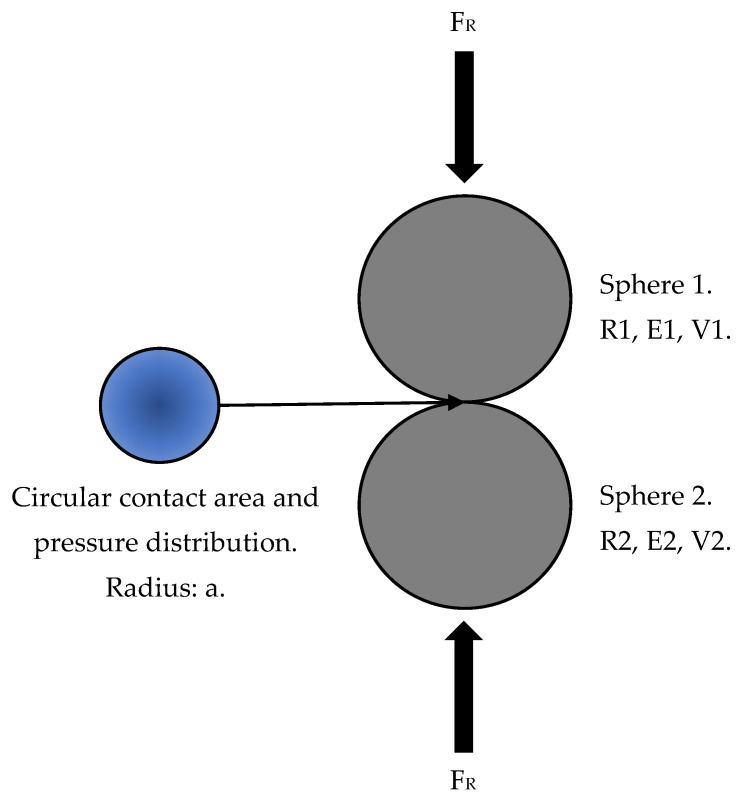
Hertzian contact between two spheres.

In theory, the contact area between two spheres or circles is a singular point; however, this would create infinite pressure between the two surfaces, and thus, immediate yielding. In reality, a small contact area is formed by the elastic deformation of each body (treated as Hertzian contact), thereby limiting the resultant stresses (Figure 2).

For the impact of two spheres, a circular contact area is formed depending on the different radii (R_1_ and R_2_), moduli of elasticity (E_1_ and E_2_), Poisson’s ratios (ν_1_ and ν_1_), and resultant force of collision (F_R_) (Table 2, Figure 2). The radius of the contact area (a) is calculated using Equation (3) [20]:

(3) $$a=\sqrt[{3}]{\frac{3F_{R}(\frac{1\;-\;{\vartheta_{1}}^{2}}{E_{1}}+\frac{1\;-\;{\vartheta_{2}}^{2}}{E_{2}})}{4(\frac{1}{R_{1}}+\frac{1}{R_{2}})}}$$

The maximum contact pressure at the centre of the contact area is calculated through Equation (4) [7,20]:

(4) $$P_{max}=\frac{3F_{R}}{2\pi a^{2}}$$

Knowing these two relationships and the properties of the spheres, this report was able to calculate the area of contact and the required force to produce a maximum Hertzian contact stress of 3 GPa.

Through substitution and simplification, Equation (3) can be rearranged into a function of F_R_, Equation (5), as presented below:

$$a=\sqrt[{3}]{\frac{3F_{R}(\frac{1\;-\;{\vartheta_{1}}^{2}}{E_{1}}+\frac{1\;-\;{\vartheta_{2}}^{2}}{E_{2}})}{4(\frac{1}{R_{1}}+\frac{1}{R_{2}})}}$$

$$a=\sqrt[{3}]{\frac{3F_{R}(\frac{1\;-\;0.26^{2}}{320\;\times \;10^{9}}+\frac{1\;-\;0.3^{2}}{210\;\times \;10^{9}})}{4(\frac{1}{6.35\;\times \;10^{-3}}+\frac{1}{6.35\;\times \;10^{-3}})}}$$

$$a=\sqrt[{3}]{\frac{3F_{R}(7.25\times 10^{-12})}{1259.84}}$$

$$a^{3}=\frac{3F_{R}(7.25\times 10^{-12})}{1259.84}$$

(5) $$F_{R}=\frac{1259.84a^{3}}{3(7.25\times 10^{-12})}$$

Through substitution and simplification, Equation (4) can also be rearranged into a function of F_R_, Equation (6), as presented below:

$$P_{max}=\frac{3F_{R}}{2\pi a^{2}}$$

$$3\times 10^{9}=\frac{3F_{R}}{2\pi a^{2}}$$

$$6\times 10^{9}=\frac{3F_{R}}{\pi a^{2}}$$

(6) $$2\times 10^{9}.\pi .a^{2}=F_{R}$$

By substituting Equation (5) into Equation (6), F_R_ can be replaced as a function of a, enabling the report to calculate the area of contact, Equation (7):

(7) $$\begin{array}{c} 2\times 10^{9}.\pi .a^{2}=\frac{1259.84a^{3}}{3(7.25\times 10^{-12})}\\ 2\times 10^{9}.\pi .a^{2}.2.174125_{10^{-11}}=1259.84a^{3}\\ \frac{2\times 10^{9}.\pi .(2.17\times 10^{-11})}{1259.84}=\frac{a^{3}}{a^{2}}=a\\ 0.0001084296651=a\;[m^{2}]\\ 1.08\times 10^{-4}=a\;[m^{2}] \end{array}$$

After calculating the area of contact between the two spheres, the report utilised Equation (4) to identify the resultant force (F_R_) necessary to produce a Hertzian contact pressure of 3 GPa.

$$P_{max}=\frac{3F_{R}}{2\pi a^{2}}$$

$$\frac{P_{max}2\pi a^{2}}{3}=F_{R}$$

$$73.87136111=F_{R}[N]$$

$$73.87=F_{R}[N]$$

**Figure 3:**
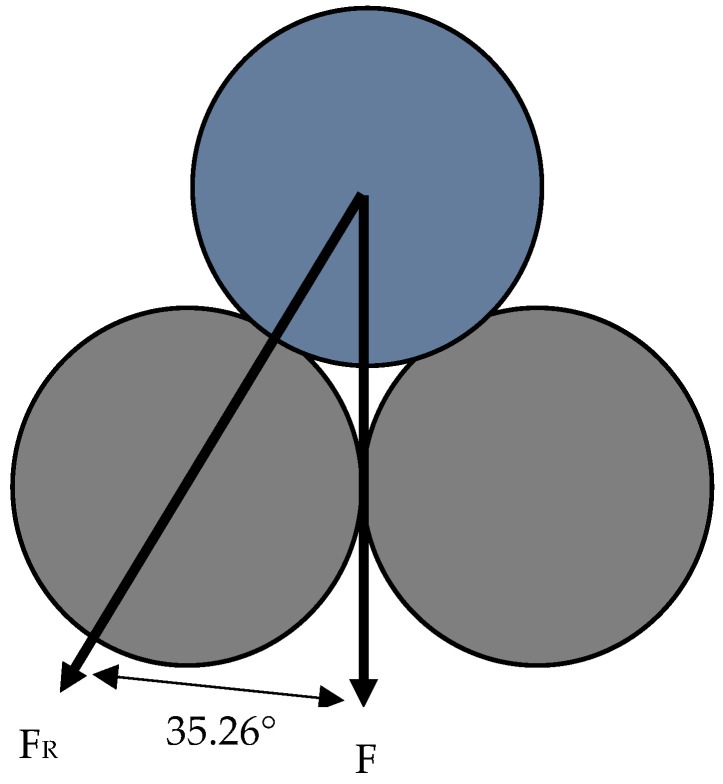
Angle of resultant force in a four-ball tribometer.

This report emphasises that these calculations are used to determine the resultant force. Comparing Figure 1 and Figure 2 shows that the resultant force within a four-ball tribometer is not parallel to the directional force applied by the machine. In fact, according to Abdullah et al. [15], the resultant force is 35.26° offset, as presented in Figure 3.

The directional force, measured in Newtons, must therefore be calculated using Equations (8) and (9):

(8) $$\begin{array}{c} F=F_{R}\cos\theta \\ F=73.87\;[N]\times \cos\left(35.26\right)\\ F=60.31786977\;[N]\\ F=60.32\;[N] \end{array}$$

Since there are three lower balls in this experiment:

(9) $$\begin{array}{c} Total\;Spindle\;Force=3F\\ Total\;Spindle\;Force=3\times 60.31786977\;[N]\\ Total\;Spindle\;Force=180.9536093\;[N]\\ Total\;Spindle\;Force=180.95\;[N] \end{array}$$

In summary, through theoretical calculations, this report was able to determine the contact area, resultant force, and spindle force required to produce a maximum Hertzian contact pressure of 3GPa and thus set the parameters and boundary conditions for both experimental and computational investigation (Table 3).

### 4.2. Finite Element Analysis

#### 4.2.1. Static Analysis

**Figure 4:**
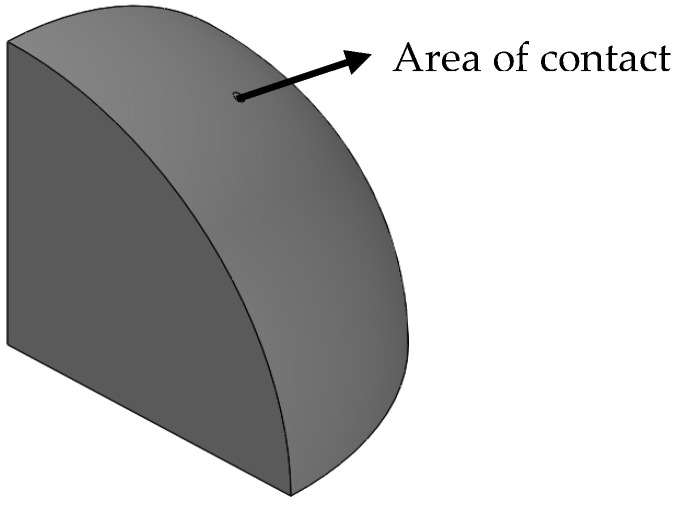
CAD model.

Using an established method for analysing localised stress [18,21], a Computer-Aided Design (CAD) model of the ceramic sphere was created, as presented in Figure 4. As shown, only an eighth segment of the sphere was created to reduce the process time of the simulation; secondly, a sketched circle (of radius a) was projected onto the outer surface at an angle of 35.26° from the central axis [15] to replicate the contact area. The designated model was then exported to SolidWorks 2023 (which is ideal for linear static and simple dynamic simulations), where detailed conditions were applied to define the scenario for numerical computation. Since this report aims to provide a working methodology for industry, the procedure is dissected further into more detailed steps.

**Figure 5:**
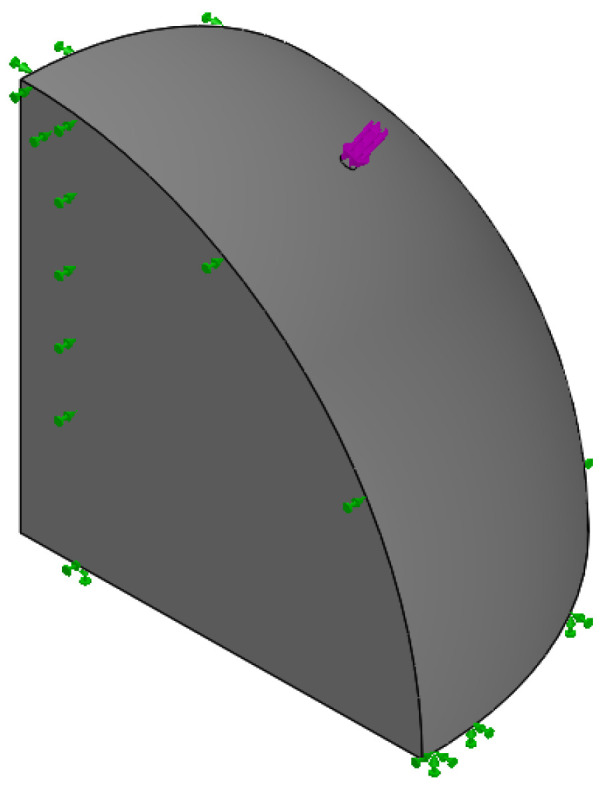
CAD model and boundary conditions.

Firstly, the material properties obtained from research and material datasheets [13,22,23] were assigned to the ceramic sphere. Cyclic symmetry fixtures were then applied to all flat faces of the CAD model to simulate the complete geometry and prevent unwanted movement in all three axes (Figure 5). This incorporated the fixing of specific nodes to inhibit any undesired translation or rotational movement [24]. A resultant force of 73.87 N was then applied to the sketched circle (projected on the curved surface) in a normal, compressive direction (Figure 5).

**Figure 6:**
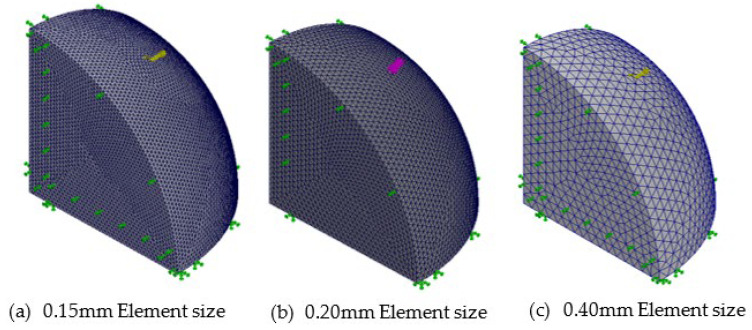
Mesh convergence analysis.

The CAD model was then discretised into smaller tetrahedra to facilitate precise convergence and thus accurate calculations. Calculating stress responses within a highly stressed volume requires high-resolution meshing to capture localised contact mechanics accurately [11]. To determine the optimum global edge length (0.20 mm) (Figure 6), a mesh convergence analysis was completed [25].

**Figure 7:**
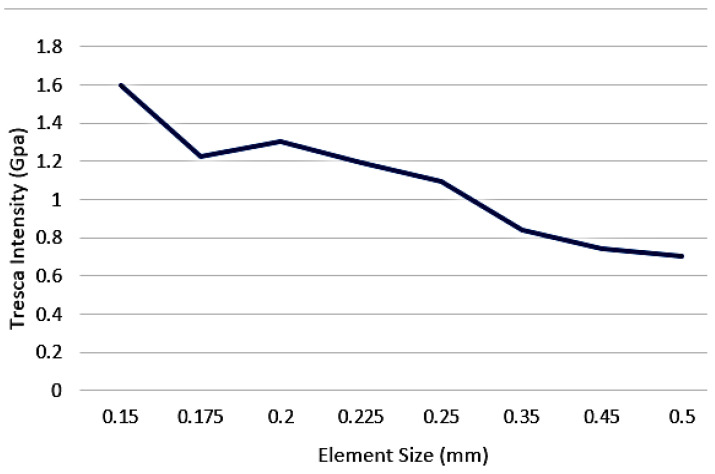
Influence of element size on Tresca intensity.

**Figure 8:**
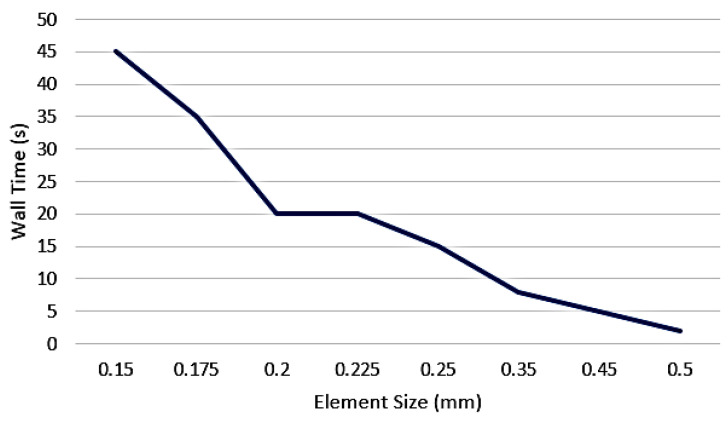
Influence of element size on wall time.

This interim investigation highlights that as the element size grows, both the Tresca intensity and wall time decrease (Figure 7 and Figure 8). However, after 0.20 mm, the element size has a reduced influence on process time and a more profound influence on results, thus identifying an element size of 0.20 mm as the optimum solution. It should be noted that, for a simple static simulation, the wall time is almost negligible (with the maximum being only 45 s), but since identical mesh techniques are used for future fatigue analysis, this report necessitates the need for optimal discretisation. Results from the static analysis were then recorded in Table 4. Tresca criterion was favoured over alternative counterparts, such as Von Mises calculations, as it defines a smaller region of elastic behaviour and is more dependent on the shear stress state, therefore providing more conservative results [26]. This is especially useful when investigating ceramic ball bearings, where designs are prone to high variability and uncertainty due to grain aspect ratios and the distribution of grain boundary phases [5].

#### 4.2.2. Fatigue Analysis

A secondary fatigue operation was set up within SWS using the previous static analysis as underlying parameters for the event. A semi-log S-N curve plotting stress amplitude against cycles to failure was obtained from Karadimas and Salonitis [12] and added to the Si_3_N_4_ material properties, whilst a zero-based loading event was applied to the sphere for 360,000 cycles. The event changes the constant 73.87 N load used beforehand to a pulsating effect with minimum and maximum values of 0 N and 78.87 N, respectively, adding a stress ratio of 0. This mimics the ceramic sphere rotating and impacting the lower three steel balls. Since the sphere is gyrating at 2000 RPM, across three balls, 360,000 cycles correspond to one hour on the tribometer. The fatigue analysis results were then transcribed into Table 4. Since fatigue is reliant on the associated stress amplitude, this report used Equation (10) [27] to help visualise the stress state within the ceramic ball.

(10) $$\begin{array}{c} Stress\;Amplitude=\frac{\left({Stress}_{Maximum}-{Stress}_{Minimum}\right)}{2}\\ \sigma_{a}=\frac{\left(\sigma_{Max}-\sigma_{Min}\right)}{2} \end{array}$$

#### 4.2.3. Lubricated Analysis

**Figure 9:**
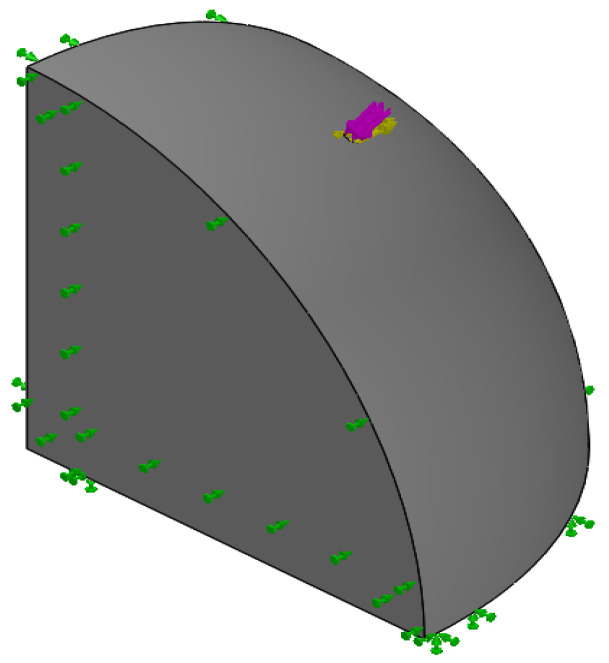
CAD model with additional boundary conditions.

In reality, when the ball bearing is rotating, friction is resisting the circular gyration, traction is transmitting friction between the spheres, and creep is occurring due to the small amount of slip between rolling surfaces [2]. An increased traction increases the peak magnitude of the maximum shear stress and drives it closer to the surface, which directly reduces rolling-contact fatigue life [11]. To incorporate these additions into the model, a secondary force was applied to the edge of the projected curve (Figure 9) in an anticlockwise direction (opposing motion). 

The magnitude of the frictional force was controlled by the coefficient of friction between the two surfaces Equation (11), as this influences all three secondary forces [2] whilst imitating a boundary lubricant. By analysing different coefficients (Table 4), this report was able to investigate the impact of different lubricants and their influence on cyclic loading, as it is commonly agreed that lubrication reduces friction between two surfaces. These setups were then implemented as underlying parameters for the events defined within fatigue investigations; all results were documented within Table 4.

It should be noted that friction coefficients approaching 1.0 are not representative of typical lubricated rolling-contact conditions and were included solely as upper-bound sensitivity cases to investigate the influence of friction on stress development and fatigue initiation.

(11) $$\begin{array}{c} Frictional\;Force=Coefficent\;of\;Friction\times Resultant\;Force\\ F_{Friction}=\mu \times F_{R}(N) \end{array}$$

## 5. Discussion

### 5.1. Static and Fatigue Analysis Discussion

**Figure 10:**
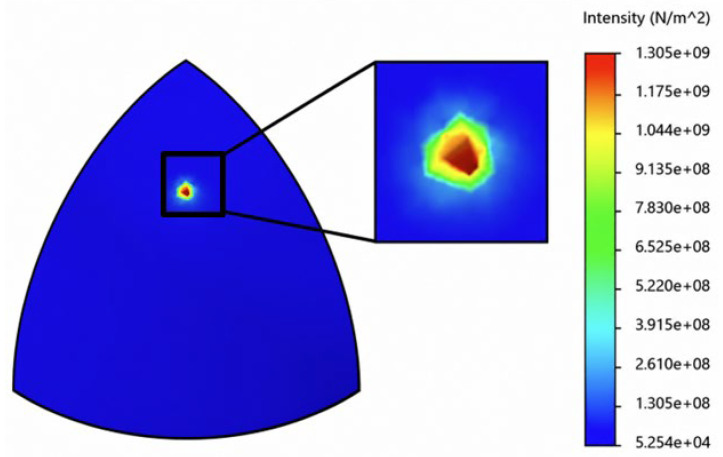
Static stress results.

When a 12.7 mm diameter Si_3_N_4_ ball bearing is subjected to a 3 GPa Hertzian contact pressure against a 12.7 mm diameter carbon chromium steel sphere, a normal force of 73.87 N is distributed across a contact area of 1.08 × 10^−4^ m^2^ (Table 3). According to the Tresca criterion, this generates a localised stress state with a maximum intensity of 1.31 GPa and a corresponding stress amplitude of 0.65 GPa (Table 4 and Figure 10). Since hot-isostatically pressed Si_3_N_4_ possesses a compressive strength of approximately 3 GPa [23] and a fatigue limit of 1.02 GPa [12], the predicted stress amplitude remains below the fatigue threshold, indicating that fatigue failure is not expected under the baseline loading conditions. This observation is consistent with the experimental findings, where no surface failure was observed during testing [13].

Beyond the numerical agreement, the finite element results provide important insight into the underlying mechanics of rolling-contact fatigue. The stress distribution is highly localised within the Hertzian contact region, producing a concentrated subsurface stress field directly beneath the loaded area, consistent with classical Hertzian contact theory [20]. Such stress localisation arises because the applied load is transmitted through a very small contact area, generating steep stress gradients and elevated shear stresses within a confined volume of material. As shown in Figure 10, the stress intensity rapidly decreases away from the contact zone, indicating that the majority of the bearing volume remains relatively unstressed. In contrast, the contact region experiences severe localised loading.

This behaviour is particularly significant for ceramic rolling elements because fatigue damage is governed by local stress concentrations rather than the nominal applied load. The pronounced subsurface stress gradients observed in the present study create favourable conditions for crack nucleation at microstructural defects, inclusions, residual stress sites, or material discontinuities [12,28,29,30].

Although the idealised model predicts no fatigue failure under the investigated loading conditions, the concentrated stress field highlights the critical role of localised deformation and cyclic stress accumulation in controlling rolling-contact fatigue performance. Furthermore, the localisation of the maximum stress beneath the contact surface supports previous observations that fatigue cracks in ceramic bearings frequently initiate within subsurface regions before propagating towards the surface under repeated loading cycles [28,29,30]. Consequently, accurate representation of Hertzian stress distributions and associated stress concentrations is essential for reliable fatigue-life prediction and for understanding the mechanisms governing crack initiation and propagation in Si_3_N_4_ rolling-element bearings.

### 5.2. Lubricated and Fatigue Analysis Discussion

**Figure 11:**
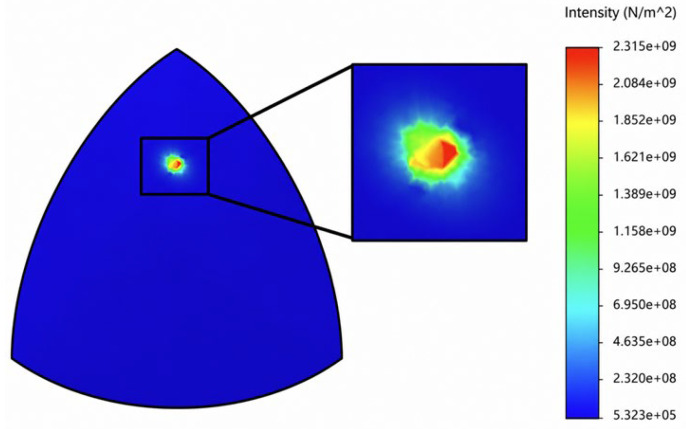
Tresca intensity at maximum friction.

The first major trend regarding lubrication is the positive correlation between the coefficient of friction and the associated frictional force (Table 4). With closer inspection, it is clear that the impact is also linear; the increase in Newtons from increasing the coefficient from 0.2 to 0.4 is nearly identical to the increase from 0.8 to 1.0 (Table 4). However, the correlation between the coefficient of friction and induced stress (Tresca intensity) is nonlinear, with increases being more influential at higher coefficients [3] (Table 4), presenting a maximum intensity of 2.32 GPa (Figure 11). Moreover, it is understood that as the normal force (resultant force) increases, so does the frictional coefficient [2] Equation (8); this is escalated by Zhang et al. [1], who claim the silicon oxide layer formed on the outside of the ceramic ball diminishes with increased pressure, exposing a rougher surface that subsequently increases friction and thus accelerates this concept.

Although practical refrigerant-lubricated contacts typically operate at substantially lower friction coefficients, the higher values considered in this study provide insight into the sensitivity of stress amplitude and fatigue life to severe lubrication degradation.

Subsequently, the same relationship is true between the coefficient of friction and stress amplitude; an increase from 0.2 to 0.4 results in an additional 0.07 GPa, whilst an increase from 0.8 to 1.0 generates an extra 0.16 GPa, over double the previous value. This is a significant finding as it is directly related to potential fatigue failure. In this case, fatigue failure was not reported until the amplitude stress exceeded 1.02 GPa (Table 4) [12,13], thus validating the FEA approach.

**Figure 12:**
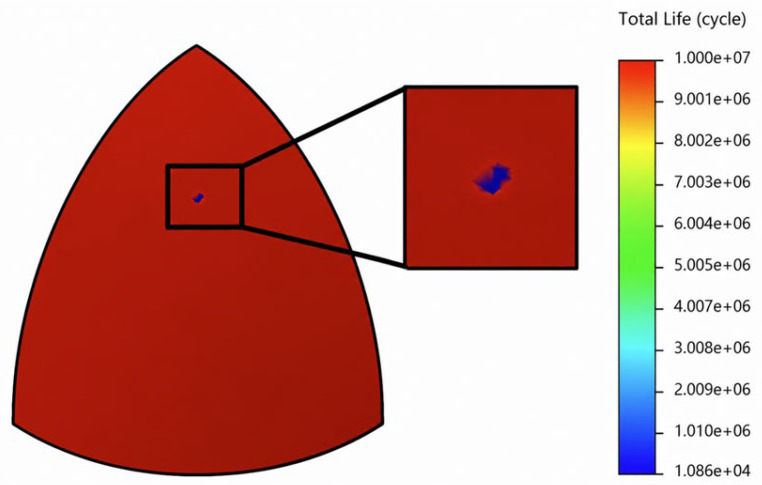
Cycles until failure at maximum friction.

When fatigue was predicted at a maximum friction of 1.0, the simulations estimated 1.086 × 10^4^ cycles, which is approximately 2 min on the four-ball rotary tribometer (Figure 12). By extrapolating this data, the report predicts that failure will commence at a coefficient of 0.83, due to the stress amplitude reaching 1.024 GPa, exceeding the fatigue limit of the material. This finding expands on current research and implies that Si_3_N_4_ ball bearings under compression of 73.87 N must have appropriate lubrication that reduces friction more effectively than artificial seawater [1], as the increasing temperature [3] may not always be viable. Moreover, a combination of high contact pressure and large friction coefficients is likely to generate a local temperature increase which would exceed the phase transformation temperature, resulting in a strong softening of the ceramic bearing and severe plastic deformation.

### 5.3. Further Analysis

**Figure 13:**
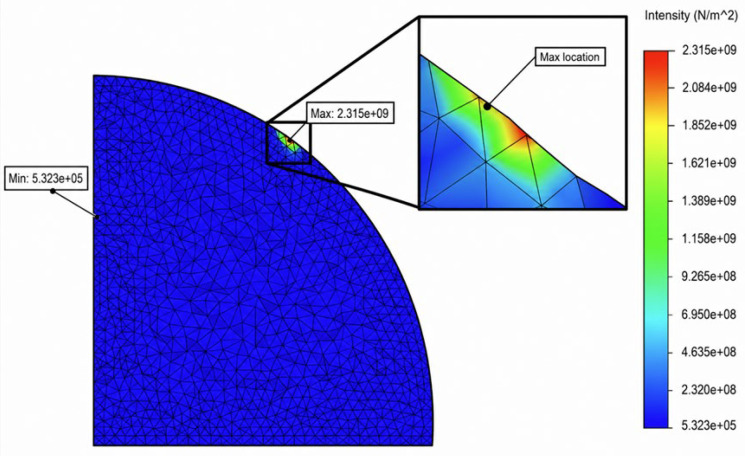
Tresca intensity at maximum friction, cross-section.

As shown by the above methodology, this report studied a perfect ball bearing with no imperfections and therefore predicted minimal deformation and no cyclic failure. However, in reality, microscopic discrepancies can often evolve into subsurface cracks within the body, which, if left untreated, can cause final rupture and ultimate failure of the mechanism [11,12]. This is significantly important as even at low friction levels, the FEA simulations recorded a stress intensity of 1.31 GPa, which far exceeds the fracture toughness of hot-isostatically pressed silicon nitride (6.5 MPam^1/2^ [23]), meaning cracks in the surrounding area are likely to propagate. As highlighted in Figure 13, the localised state has a hemispherical volume with a radius of 0.11mm originating at the point of contact (depth of 0.11 mm, breadth of 0.22 mm), with the stress intensity dissipating towards the periphery. Overall, this creates a 0.005 mm^3^ region for plausible crack growth. Furthermore, due to rolling motion, this region will be extended along the periphery of the bearing in a strip otherwise known as the contact track [15]. Meanwhile, some scholars advise adding artificial cracks to the models [28,29,30].

This is inappropriate for a general predictive method, as defects will vary indefinitely from sphere to sphere, and it is unlikely that manufacturers will knowingly produce severely defective bearings. To compensate, the predicted fatigue life should be interpreted within the framework of established bearing-design standards, with safety margins selected according to the specific operating conditions and reliability requirements of the intended application.

## 6. Conclusions

The present study developed and validated a finite element methodology for predicting rolling-contact fatigue in hot-isostatically pressed Si_3_N_4_ ball bearings operating under lubricated Hertzian contact conditions. The principal findings are summarised as follows:

○ A Hertzian contact pressure of 3 GPa generated a maximum contact force of 73.87 N and a peak Tresca stress of 1.31 GPa, corresponding to a stress amplitude of 0.65 GPa, which remained below the reported fatigue limit of Si_3_N_4_ (1.02 GPa).
○ The numerical predictions were consistent with the experimental observations reported by Khan [13], where no fatigue-related surface failure was observed after 3.83 × 10^6^ cycles, confirming the validity of the proposed finite element framework.
○ Friction was identified as the dominant parameter controlling fatigue performance. Increasing the coefficient of friction from 0 to 1.0 increased the maximum Tresca stress from 1.31 to 2.32 GPa and increased the stress amplitude from 0.65 to 1.16 GPa.
○ Fatigue failure was not predicted for friction coefficients up to 0.8. Failure occurred only when the friction coefficient reached 1.0, producing an estimated fatigue life of 1.086 × 10^4^ cycles.
○ Extrapolation of the numerical results indicates a critical friction coefficient of approximately 0.83, above which the stress amplitude exceeds the fatigue endurance limit of the ceramic material.
○ The original hypothesis that lubrication can significantly improve rolling-contact fatigue performance was confirmed. The results demonstrate that reducing friction maintains the stress amplitude below the endurance limit, whereas high-friction conditions rapidly accelerate fatigue damage accumulation.
○ Compared with previous studies that primarily focused on experimental fatigue testing [13,28,29,30] or reliability-based optimisation approaches [16], the present work provides a computationally efficient framework capable of linking Hertzian contact loading, frictional behaviour, lubrication effects and fatigue life prediction within a single modelling methodology.
○ The results provide direct guidance for the development of lubricants and lubricant additives for ceramic rolling bearings operating under severe conditions. Additive technologies should prioritise maintaining low friction coefficients through the formation of stable tribofilms, suppression of surface roughening, and reduction in rolling–sliding interactions.
○ The numerical results indicate that maintaining friction coefficients below approximately 0.8 is essential for preventing the stress amplitude from exceeding the fatigue limit of Si_3_N_4_. Consequently, lubricant formulations that preserve low-friction interfaces under elevated contact pressures are expected to significantly enhance bearing reliability and service life.

## Figures and Tables

**Table 1:** Experimental conditions and results adapted from Khan [13].

Specimen	Hertzian Contact Stress (GPa)	Rolling Velocity (m s^−1^)	Lubricant	Chamber Environment	Time (min)	Stress Cycles (×10^6^)	Comment
1	3.0	1.33	R600a (isobutane)	Pressurised chamber	284	1.28	Suspended due to no surface failure
2	3.0	1.33	R600a (isobutane)	Pressurised chamber	850	3.83	Suspended due to no surface failure

**Table 2:** Sphere properties.

Sphere No.	Material	Radius (m)	Elastic Modulus (Pa)	Poisson’s Ratio (ϑ)
1	Si_3_N_4_	6.35 × 10^−3^	320 × 10^9^	0.26
2	Carbon chromium steel	6.35 × 10^−3^	210 × 10^9^	0.30

**Table 3:** Overview of theoretical calculations.

The Radius of the Contact Area (m^2^)	Resultant Force (N)	Total Spindle Force (N)
$1.08\times 10^{-4}$	73.87	180.95

**Table 4:** Simulation results.

**Static Analysis with Fatigue Results**					
Coefficient of Friction	Normal Force (N)	Frictional Force (N)	Tresca Intensity (GPa)	Stress Amplitude (GPa)	Fatigue Cycles (×10^4^)
-	73.87	-	1.31	0.65	No Fatigue
**Lubricated Analysis with Results**					
Friction Coefficient	Normal Force (N)	Frictional Force (N)	Tresca Intensity (GPa)	Alternating Stress (GPa)	Fatigue Cycles
0.00	73.87	0.00	1.31	0.65	No Fatigue
0.20	73.87	14.77	1.36	0.68	No Fatigue
0.40	73.87	29.55	1.49	0.75	No Fatigue
0.60	73.87	44.32	1.71	0.86	No Fatigue
0.80	73.87	59.10	2.00	1.00	No Fatigue
1.00	73.87	73.87	2.32	1.16	1.086

## Data Availability

The original contributions presented in this study are included in the article. Further inquiries can be directed to the corresponding author.
